# Analysis and Experimental Validation of Rheumatoid Arthritis Innate Immunity Gene CYFIP2 and Pan-Cancer

**DOI:** 10.3389/fimmu.2022.954848

**Published:** 2022-07-11

**Authors:** ZhenYu Zhao, ShaoJie He, XinCheng Yu, XiaoFeng Lai, Sheng Tang, El Akkawi Mariya M., MoHan Wang, Hai Yan, XingQi Huang, Shan Zeng, DingSheng Zha

**Affiliations:** ^1^Department of Orthopaedics, The First Affiliated Hospital, Jinan University, Guangzhou, China; ^2^Department of Orthopaedics, Panyu Hospital of Chinese Medicine, Guangzhou, China; ^3^Department of Orthopedics, The Sixth Affiliated Hospital, South China University of Technology, Foshan, China; ^4^Department of Plastic and Reconstructive Surgery, ZhuJiang Hospital of Southern Medical University, GuangZhou, China; ^5^Department of Medicine, Flushing Hospital Medical Center, Flushing, NY, United States; ^6^Department of Neurosurgery , General Hospital of Tianjin Medical University, China; ^7^Department of Rheumatology, The First Affiliated Hospital, Jinan University, Guangzhou, China

**Keywords:** rheumatoid arthritis, CYFIP2, GEO, WGCNA, pan-cancer, ST8SIA1, CIA mouse

## Abstract

Rheumatoid arthritis (RA) is a chronic, heterogeneous autoimmune disease. Its high disability rate has a serious impact on society and individuals, but there is still a lack of effective and reliable diagnostic markers and therapeutic targets for RA. In this study, we integrated RA patient information from three GEO databases for differential gene expression analysis. Additionally, we also obtained pan-cancer-related genes from the TCGA and GTEx databases. For RA-related differential genes, we performed functional enrichment analysis and constructed a weighted gene co-expression network (WGCNA). Then, we obtained 490 key genes by intersecting the significant module genes selected by WGCNA and the differential genes. After using the RanddomForest, SVM-REF, and LASSO three algorithms to analyze these key genes and take the intersection, based on the four core genes (BTN3A2, CYFIP2, ST8SIA1, and TYMS) that we found, we constructed an RA diagnosis. The nomogram model showed good reliability and validity after evaluation, and the ROC curves of the four genes showed that these four genes played an important role in the pathogenesis of RA. After further gene correlation analysis, immune infiltration analysis, and mouse gene expression validation, we finally selected CYFIP2 as the cut-in gene for pan-cancer analysis. The results of the pan-cancer analysis showed that CYFIP2 was closely related to the prognosis of patients with various tumors, the degree of immune cell infiltration, as well as TMB, MSI, and other indicators, suggesting that this gene may be a potential intervention target for human diseases including RA and tumors.

## Introduction

RA is a chronic, symmetrical, autoimmune disease that is aggressive and involves multiple joints in the body. The worldwide prevalence is approximately 5 per 1,000 and the incidence in women is usually 2 to 3 times higher than in men (1). RA is characterized by painful, morning stiffness, which leads to joint erosion and destruction, producing limb deformities. Some patients with RA may or subsequently develop manifestations involving organs other than joints, such as rheumatoid nodules in the skin, pericarditis, and interstitial lung lesions (2), making RA a multisystem disease. The diagnosis of RA is mainly based on clinical symptoms, signs, and laboratory and imaging tests. Therefore, it is easy to miss the diagnosis of early, atypical, or inactive RA. Recently, large-scale genome-wide association studies (GWAS) and meta-analyses have revealed common disease-associated variants in the population, and there is an increasing number of studies on genes and susceptibility to RA, increasing the possibilities for early diagnosis and clinical treatment of RA (3).

The etiology and pathogenesis of RA are complex, and the immune response occurs under the combined effect of multiple factors influenced by genetics, infection, and environment, causing synovitis. Studies have shown that the abnormal morphology and gene expression patterns of RA synovial fibroblasts (RASF) and macrophages (RASM) are key factors in the development of RA (4, 5). B cells secrete proteins such as rheumatoid factor (RF), anti-citrullinated protein antibodies (ACPA), and pro-inflammatory cytokines to form immune complexes with self-antigens to support RA (6).T cells differentiate into TH1, TH17, or Tfh and release lymphokines. In RA, the main function of T cells is to activate macrophages and fibroblasts, which differentiate into tissue-damaging cells (7). However, the mechanisms of gene and protein expression in the synovium associated with the pathogenesis of RA have not been elucidated.

The main treatments for RA are anti-inflammatory drugs, analgesic drugs, and DMARDs.The first two can only relieve the symptoms of RA but do not stop the further development of RA. Since the immune response is the main pathogenesis of RA, disease-modifying antirheumatic drugs (DMARDs) have become the primary choice for RA. Although DMARDs have shown good efficacy in reducing RA, there is still a possibility of treatment failure with DMARDs for some patients (8). In the last decade, biologics have continued to enter clinical trials, and these drugs specifically target immune cells for immunomodulation and are used in conjunction with DMARDs for the treatment of RA (9). Whether DMARDs are used alone or in combination with new biologic agents, the optimal therapeutic options for RA are still under further investigation. Therefore, there is an urgent need to explore the signature genes that are closely related to the development of RA in order to provide better options for early diagnosis and treatment of RA.

In this article, we used the R tool and the limma package to statistically analyze the four data sets and analyze the differential expression of mRNAs. The WGCNA R package was then used to calculate the association of gene significance (GS) and module membership (MM), analyze the correlation between modules to construct a weighted gene co-expression network, and merge DEGs with key module genes for functional analysis. The feature genes were also identified by the algorithm, and LASSO regression analysis was performed to narrow down the range of feature genes. To further validate the selected signature genes, we used GSEA analysis, interaction analysis, ROC analysis, and studied the level of immune cell infiltration in the RA group, and finally calculated the relationship between signature genes and immunity. For further selection, by integrating multiple datasets, we aimed to screen the signature genes that play a key role in the development of RA and various cancers. Combining with the immune infiltration analysis and *in vivo* experiment, CYFIP2 was filtered out and verified *via* pan-cancer analysis, which illustrated a strong correlation with various tumors.

## Material and Methods

### Data Processing and Download of the RA Dataset

GSE1919 (10), GSE55457 (11), GSE48780 (12), and GSE55235 (11) were downloaded from the Gene expression omnibus (Geo, https://www.ncbi.nlm.nih.gov/geo/) database, and information on these datasets is supplied in **Table 1**. “Limma” software was used to investigate mRNA expression differences (13). To account for false-positive results, adjusted P-values were examined in GEO. The R package ggord was used to depict the threshold mRNA differential expression screen, which was specified as “Adjusted P <0.05 and log2 (fold change) >0.5 or log2 (fold change)<−0.5.” The R package pheatmap was used to create the expression heat maps. From the InnateDB database, a total of 2,308 immune genes involved in the innate immune response were obtained. The Cancer Genome Atlas provided RNA sequencing and clinical data for 33 different cancer types (TCGA). The GTEx database was used to collect normal tissue expression data, while the CCLE database was used to obtain the gene expression for several cancer cell lines.

**Table 1:** Information on microarray datasets obtained from Gene Expression Omnibus.

GEO Data set	Platform	RA	Control
GSE1919	GPL91	5	5
GSE55457	GPL96	13	10
GSE48780	GPL570	83	0
GSE55235	GPL96	10	10

### Enhancement of Functionality

The data were evaluated using functional enrichment to confirm the possible functionalities of prospective targets. Gene ontology (GO) is a popular technique for assigning functions to genes, particularly molecular functions (MF), biological pathways (BP), and cellular components (CC). KEGG enrichment analysis can be used to analyze gene functions as well as related high-level genomic functional information. The “GOplot” package and the “cluster profiler” in R were used to examine the GO function of prospective mRNAs and to enhance KEGG pathways to better understand the carcinogenic role of target genes (14).

### Co-Expression Networks are Built

The WGCNA method aids in the investigation of gene set expression. Through the following main phases, the WGCNA R package was used at various stages for the development and modularization of various gene networks. To determine if there were any significant outliers, the samples were placed in clusters. Following that, automated networks were used to create co-expression networks. The modules used hierarchical clustering and dynamic tree cutting function detection. Module membership (MM) and gene significance (GS) were estimated to connect modules with clinical characteristics. Hub modules were designated as those with the highest Pearson module membership correlation (MM) and a p absolute value of 0.05. High module connection and clinical importance were denoted by MM >0.8 and GS >0.2, respectively. The gene information for the corresponding module was advanced for further investigation (15).

### Identification of Distinct Genes

The above genes were used to isolate the feature genes that were used to diagnose RA. SVM is a regression or classification-supervised machine learning technique that requires a training set with labels (16). SVM-RFE is a machine learning technique that trains a subset of features from different categories to shrink the feature set and find the most predictive features. To compute and choose linear models and keep the valuable variables, the “glmnet” package in R was used to perform minimum absolute shrinkage and selection operator (LASSO) regression. The binomial distribution variables were then used in the LASSO classification, coupled with one standard error lambda value for the minimum criterion (1−SE criterion) used to build the model, which has good performance but only 10 cross-validation variables. RandomForest was used to rank the genes, and their relative value above 0.25 was recognized as a typical chance cause (17). The intersection was then used to pick the most significant feature genes in this study using LASSO logistic regression, SVM-RFE, and RandomForest.

### PPI (Protein–Protein Interaction) Network Construction

GeneMANIA (http://www.genemania.org) is a website for building protein–protein interaction (PPI) networks, which can be used to generate gene function predictions and locate genes with comparable effects. Physical interaction, co-expression, co-localization, gene enrichment analysis, genetic interaction, and site prediction are some of the bioinformatics methods used by the network integration algorithm. GeneMANIA was used to analyze PPI networks of signature genes in this study.

### Diagnostic Column Line Graph Construction and Validation

We created a column line graph model to predict the recurrence of RA using the “rms” program. The “score” is the score of the relevant item below, and the “total score” is the sum of all the elements above. The predictive power of the line graph model was then assessed using calibration curves. Finally, decision curve analysis and clinical impact curves were used to assess the clinical utility of the model.

### Curve Analysis of Receiver Operating Characteristics (ROC)

We used the P ROC function in the R package to create Receiver Operating Characteristic (ROC) curves to determine the area under the curve (AUC) for screening signature genes and evaluating their diagnostic value (18).

### Immune Infiltration Analysis by ssGSEA

To investigate the various levels of infiltration of immune cell types between RA tissue and normal tissue. To analyze the association between immune cells and distinctive genes, the “corrplot” package was used to obtain the Spearman rank correlation coefficient.

### Analysis of Prognosis

Using deep forest plots, the “foresrplot” R program was used to perform univariate cox regression analysis and display p-values, HRs, and 95% CIs.

### Analysis of Immune Infiltration

We used TIMER, XCELL, QUANTISEQ, MCPCOUNTER, and EPIC algorithms to explore the relationship between AXIN1 expression and immune invasion in all TCGA tumors.

### For Systematic Collagen-Induced Arthritis (CIA) Mouse Setup, HE Staining, and IHC

The Animal Care & Ethics Committee of Jinan University’s First Hospital approved all animal care and experimental operations. We also followed the Guide for the Care and Use of Laboratory Animals that was established by the National Institutes of Health. The ARRIVE criteria were followed for reporting animal experiments (19, 20). Mice (n = 22) were given 200 g of bovine type II collagen (Sigma, St. Louis, MO, USA), diluted in acetic acid, and emulsified at a 1:1 ratio (vol/vol) in Forster’s complete adjuvant intradermally at the tail vein. Mice were booster-immunized three weeks after the initial immunization with a 1:1 ratio (vol/vol) intraperitoneal injection of bovine type II collagen emulsified in incomplete Freund’s adjuvant. From days 32 to 41 after the initial immunization, episodes of illness characterized by erythema and/or paw edema were seen. As previously described (21), mice were checked daily for indications of arthritis, and the severity of arthritis was graded on a scale of 0 to 3. The arthritis scores of the mice were determined for all four paws. The dimensions of the ankle joints were measured with 0.01 mm accuracy with vernier calipers. All mice were given 110 mg/kg ketamine and 4.8 mg/kg xylazine before having their hind limbs amputated and fixed in 10% neutral buffered formalin. Tissues were decalcified in 8% formic acid and paraffin-embedded. Hematoxylin and eosin were used to stain 3 mm sections (H&E). A previously established scoring system was used to calculate inflammation rates (21).

### Immunohistochemistry

For immunohistochemistry, synovial tissue sections were stripped and then incubated with 5% serum in PBS for 2 h to block nonspecific binding and with 3% H2O2 for 10 min to block endogenous peroxidase activity. The expression of CYFIP2 and ST8SIA1 was determined by staining with polyclonal rabbit anti‐mouse CYFIP2 and ST8SIA1 antibodies overnight at 4°C. As controls, irrelevant isotype-matched antibodies were used. A polyclonal goat anti-rabbit antibody was detected with diaminobenzidine using goat anti-rabbit antibodies labeled with HRP.

### Data and Statistical Analysis

Data collection and analysis complied with pharmacology’s recommendations for experimental design and analysis (22). The *in vitro* experiments were conducted with a minimum of five independent experiments. Therefore, the results were expressed as mean + SEM. Blinding was used in the experimental procedures or treatment and data analysis. We normalized immunoblots, glucose uptake, and mRNA expression for quantitative analysis to reduce baseline variations between independent experiments. Comparing the two groups was done using Student’s t-test. The one-way ANOVA was applied to three or more different groups. If F exceeded 0.05 and the variance in homogeneity was not significant, all results were discarded. Two *post hoc* tests were applied: Dunnett’s *post hoc* test on each group compared with the control group or Sidak’s *post hoc* test on multiple groups compared together. To analyze the data normalization, a non-parametric statistical analysis was performed. Data with non-parametric characteristics were analyzed with the Kruskal-Wallis test or Wilcoxon test two-sample. The statistical analyses of the data were conducted using SPSS 13.0 software. A P-value of less than 0.05 was considered significant.

## Results

### DEG Screening and Data Preprocessing

**Figure 1:**
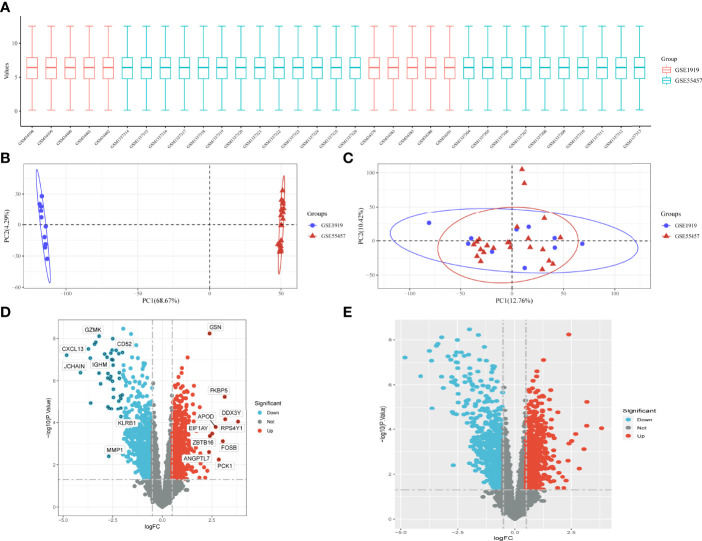
Data preprocessing for DEG. **(A)** Box plots of raw data normalized between samples. **(B, C)** PCA of RA and control samples. **(D)** Volcano plot of DEG. **(E)** Heat map of DEG.

The data are standardized in a box plot, where different colors represent different data sets, rows represent samples, and columns represent gene expression values in samples (**Figure 1A**). **Figure 1B** depicts the PCA results of multiple data sets before batch removal are displayed, where different colors represent different data sets. As shown in the diagram, three data sets are separated separately without any intersection. **Figure 1C** shows the PCA result diagram after batch removal. As shown in the diagram, the intersection of three data sets can be used as a batch of data for subsequent analysis. Under the criteria of P-adjustment <0.05 and log2 fold-change (FC) | >0.5, 891 genes were identified as DEGs, with 427 genes up-regulated and 464 genes down-regulated. **Figure 1D** shows volcano plots of DEGs as well as a heat map of the top 50 genes (**Figure 1E**).

### DEGs Functional Enrichment Analysis

All DEGs were functionally enriched, and 15 GO keywords were exhibited in the GOCircle plot according to p <0.05 (**Supplementary Figure 1A**, **Supplementary Table1**). The findings revealed that the biological process (BP) enrichment was primarily connected to the positive cell–cell adhesion regulation, T-cell activation, lymphocyte differentiation, and cell–cell adhesion regulation. Enriched molecular function (MF) is related to cytokine receptor binding, cytokine binding, and cytokine receptor activity. Cellular component (CC) enrichment is related to the external side of the plasma membrane, membrane raft, and membrane microdomain. Hematopoietic cell lineage, Human T-cell leukemia virus 1 infection, Th1 and Th2 cell differentiation, and the chemokine signaling pathway were linked in KEGG analysis (**Supplementary Figure 1B**, **Supplementary Table 2**).

### Weighted Gene Co-Expression Network Construction

**Figure 2:**
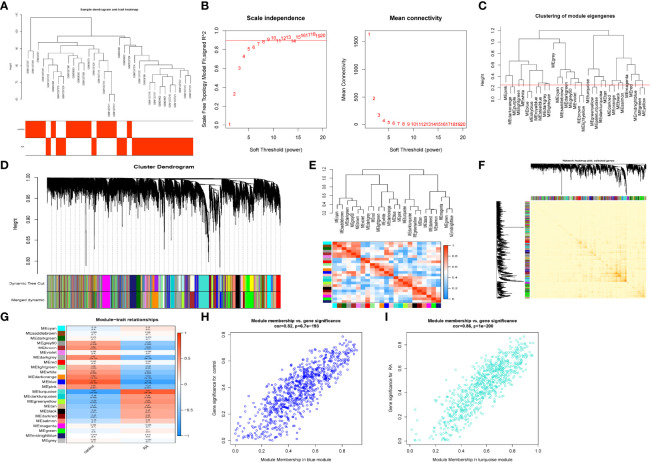
Construction of WGCNA co–expression network. **(A)** Sample clustering dendrogram with tree leaves corresponding to individual samples. **(B)** Soft threshold β = 7 and scale–free topological fit index (R2). **(C)** Clustered dendrograms were cut at a height of 0.25 to detect and combine similar modules. **(D)** Shows the original and combined modules under the clustering tree. **(E)** Collinear heat map of module feature genes. Red color indicates a high correlation, blue color indicates opposite results. **(F)** Clustering dendrogram of module feature genes. **(G)** Heat map of module–trait correlations. Red represents positive correlations and blue represent negative correlations. **(H)** MM vs. GS scatter plot of control. **(I)** MM vs. GS scatter plot of RA.

The GSE1919 and GSE55457 datasets were retrieved from the GEO data, and 15 normal samples and 18 RA samples were preferred to cluster the samples and exclude the obviously aberrant samples by setting a threshold, as shown in **Figure 2A**. Then, as shown in **Figure 2B**, we set the soft threshold to 7 when R^2^ >0.9 and the average connectivity is high. After merging the strongly associated modules using a 0.25 clustering height limit (**Figure 2C**), 24 modules were identified for further study. The primed and merged modules were eventually displayed under the clustering tree (**Figure 2D**). The correlation between modules was examined next, and the results revealed that there was no significant association between them (**Figure 2E**). The reliability of module delineation was demonstrated by transcription correlation analysis within modules, which revealed no substantial linkage between modules (**Figure 2F**). The frontal correlations between ME values and clinical features were used to investigate the link between modules and clinical symptoms. The blue module was positively correlated with normal (r = 0.79, p = 5e−08) and negatively linked with RA (r = −0.79, p = 5e−0.8), while the turquoise module was negatively connected with normal (r = 0.8, p = 3e−08) and positively correlated with RA (r = −0.8, p = 3e−08) (**Figure 2G**). Clinically meaningful modules were identified. The results showed that blue and turquoise modules were highly linked with RA in the control MM versus GS scatter plot (**Figure 2H**) and the RA MM versus GS scatterplot (**Figure 2I**). All the genes in the two modules were examined further.

### DEGs and Functional Analysis of Critical Module Genes

**Figure 3:**
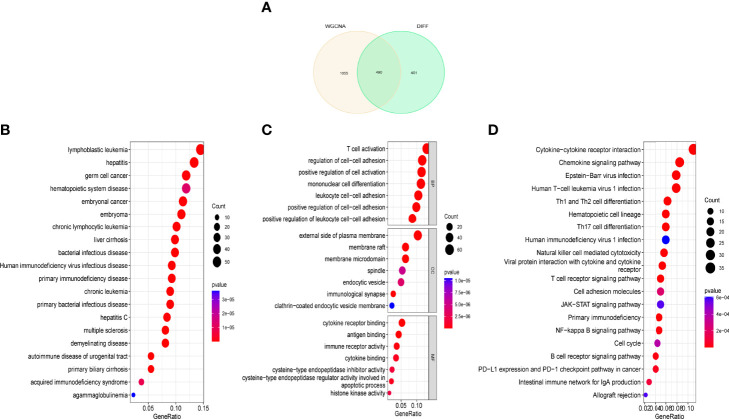
Functional analysis of key module genes merged with DEGs. **(A)** Venn diagram of key module genes versus DEGs. **(B)** DO analysis. **(C)** GO analysis. **(D)** KEGG analysis.

After overlapping critical module genes and DEG genes using a Venn diagram, we discovered 490 overlapping genes (**Figure 3A**). We performed functional analysis to learn more about the biological functions of the DEG genes in the modules. The results of DO analysis revealed that these DEGs were linked to lymphoblastic leukemia, hepatitis, germ cell cancer, and hematopoietic system disease (**Figure 3B**). GO enrichment analysis showed that module DEG genes have T-cell activation, regulation of cell-cell adhesion, positive regulation of cell activation, the external side of the plasma membrane, membrane raft, membrane microdomain, cytokine receptor binding, antigen binding, and immune receptor activity (**Figure 3C**). KEGG analysis was associated with cytokine–cytokine receptor interaction, chemokine signaling pathway, and human immunodeficiency virus type 1 infection (**Figure 3D**).

### Selection of Feature Genes

**Figure 4:**
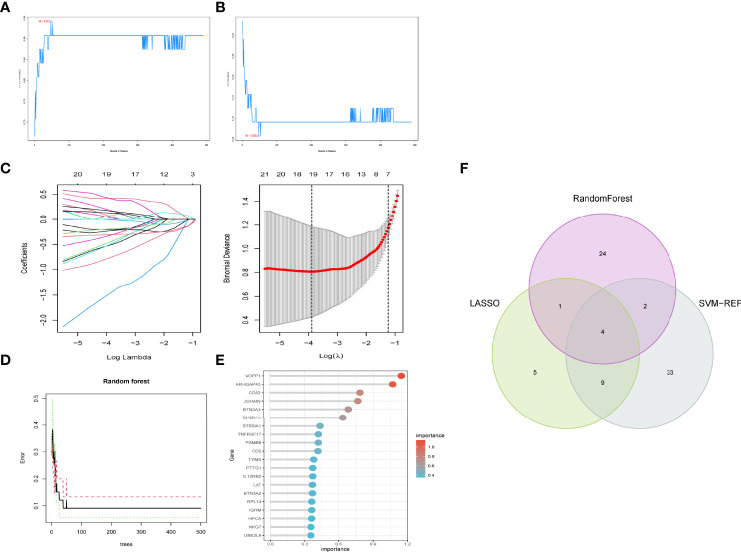
Feature gene selection. **(A,B)** Biomarker signature gene expression validation by support vector machine recursive feature elimination (SVM–RFE) algorithm selection. **(C)** Adjustment of feature selection in the minimum absolute shrinkage and selection operator model (lasso). **(D)** randomForest error rate versus the number of classification trees. **(E)** The top 20 relatively important genes. **(F)** Three algorithmic Venn diagram screening genes.

We used three machine algorithms to identify feature genes: SVM-RFE (**Supplementary Table 3**) (**Figures 4A, B**); LASSO regression analysis to select 19 predicted genes from statistically significant univariate variables (**Figure 4C**) (**Supplementary Table 4**); and RandomForest combined with feature selection to determine the relationship between error rate, number of classification trees (**Figures 4D, E**) (**Supplementary Table 5**) and 31 genes with relative importance. We used a Venn diagram to find four genes that overlapped using the intersection of the three methods discussed above (**Figure 4F**).

### Validation of Specific Gene Expression

**Figure 5:**
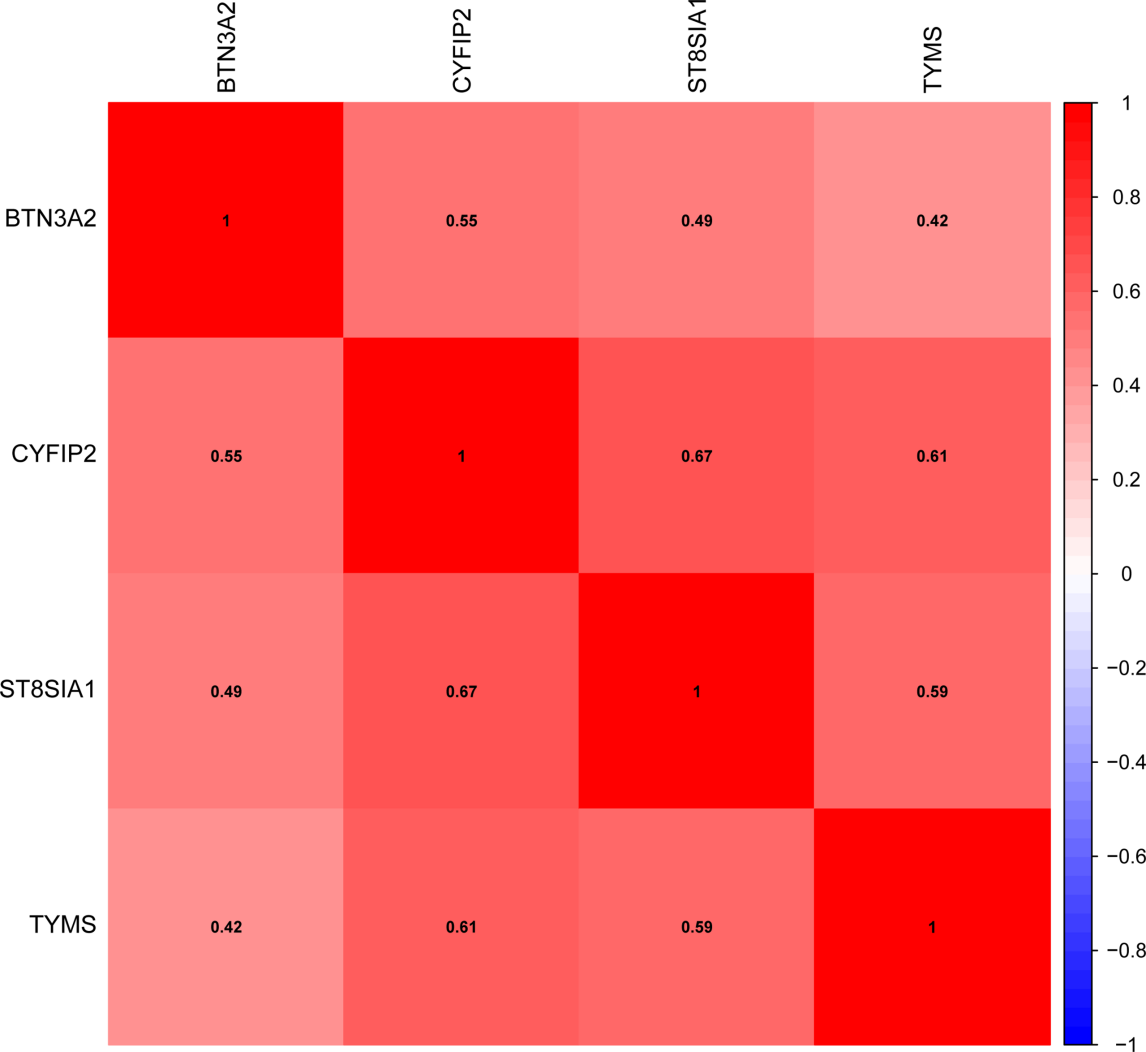
Correlation between trait genes.

We confirmed the expression of these four genes in RA using GSE1919 and GSE55447 data and found that BTN3A2, CYFIP2, ST8SIA1, and TYMS were all substantially elevated in RA **(Supplementary Figure 2A**). Additionally, validation datasets (GSE48780 and GSE55235) indicated that BTN3A2, CYFIP2, ST8SIA1, and TYMS were substantially expressed in RA (**Supplementary Figure 2B**). Gene correlations were also examined, as shown in **Figure 5**, BTN3A2, ST8SIA1, TYMS, and CYFIP2 were positively correlated, indicating that the four genes had a significant functional similarity.

### Analysis of the Feature Genes Using GSEA

To better understand the role of signature genes in RA, we used GSEA to classify RA tissues into two categories based on the median expression of signature genes. Nucleotide metabolism, primary immunodeficiency, pyrimidine metabolism, and retinol metabolism were significantly enriched in the high BTN3A2 subgroup, whereas aldosterone-regulated sodium reabsorption, and HIF-1 signaling pathway, nitrogen metabolism, and renal cell carcinoma were significantly enriched in the low BTN3A2 subgroup (**Supplementary Figure 3A**). Cocaine addiction, glycerolipid hematopoietic cell lineage,i immune network for production, and primary immunodeficiency were significantly enriched in the high CYFIP2 subgroup, whereas allograft rejection, the intestinal immune network for IgA production, nicotinate and nicotinamide metabolism, and primary immunodeficiency were significantly enriched in the low CYFIP2 subgroup (**Supplementary Figure 3B**). Ferroptosis, linoleic acid metabolism, nitrogen hematopoietic cell lineage, intestinal immune network for IgA production, primary immunodeficiency, Th1 and Th2 cell differentiation were significantly enriched in the high ST8SIA1 subgroup, while ferroptosis, linoleic acid metabolism, nitrogen hematopoietic cell lineage, intestinal immune network for IgA production, primary immunodeficiency, Th1 and (**Supplementary Figure 3C**). The high TYMS subgroup was highly enriched in immunodeficiency, Th1 and Th2 cell differentiation, whereas the low TYMS subgroup was significantly enriched in ABC transporters, circadian rhythm, glycolysis/gluconeogenesis, and proximal tubule bicarbonate reclamation (**Supplementry Figure 3D**).

### Trait Gene Interaction Analysis

**Figure 6:**
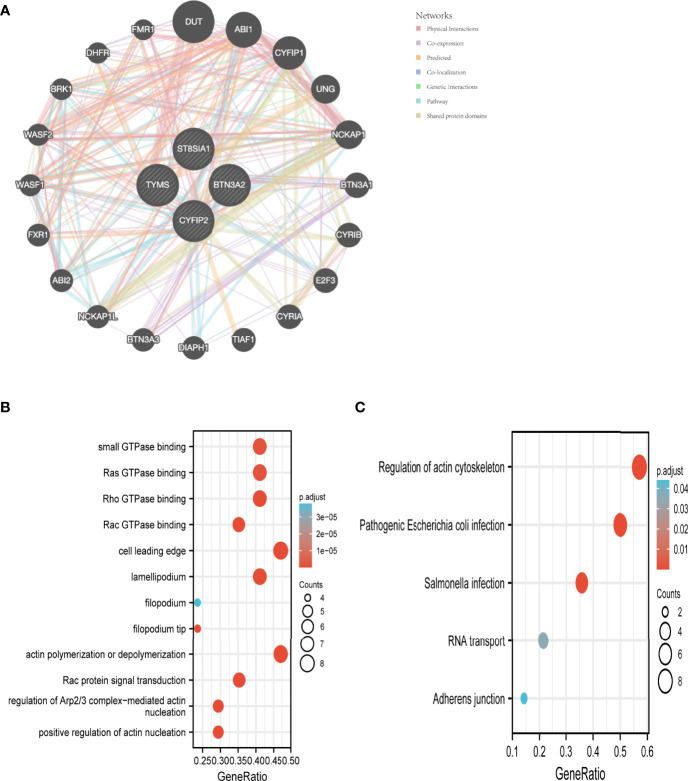
Interaction analysis of feature genes. **(A)** Characterized gene co–expression network. **(B)** GO analysis of co–expressed genes. **(C)** Co–expressed gene KEGG analysis.

We used the GeneMANIA database to create a PPI network for the signature genes (**Figure 6A**). To further investigate the function of the signature genes, GO/KEGG analysis was performed on 20 genes. Actin polymerization or depolymerization, Rac protein signal transduction, and control of Arp2/3 complex-mediated actin nucleation were the most abundant biological processes in this dataset. The cell leading edge, lamellipodium, and filopodium were the most abundant cellular components (CC). Furthermore, Rho GTPase binding, Ras GTPase binding, small GTPase binding, and Rac GTPase binding were connected to the enriched molecular functions (MF) (**Figure 6B**). The main enriched pathways, according to KEGG analysis, were the regulation of the actin cytoskeleton, pathogenic *Escherichia coli* infection, and Salmonella infection (**Figure 6C**).

### Modeling and Testing of a RA Diagnostic Column Line Graph

**Figure 7:**
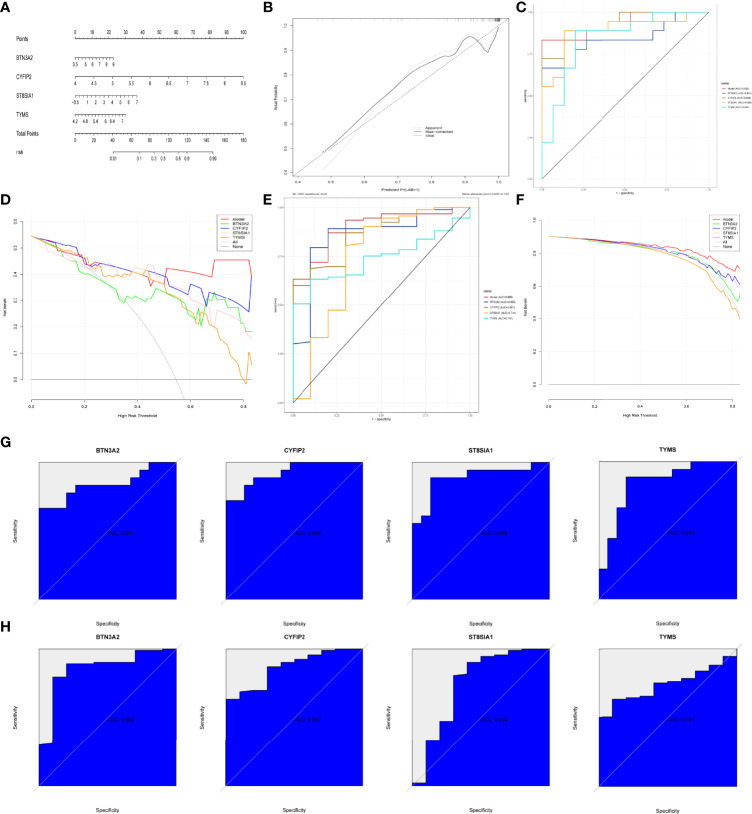
Construction and validation of the RA diagnostic column line graph model. **(A)** Column line graphs are used to predict the occurrence of RA. **(B)** ROC curves to assess the clinical value of the column line graph model. **(C)** Calibration curves to assess the predictive power of the column line graph model. **(D)** DCA curves to assess the clinical value of the column line graph model. **(E, F)** Validation set to verify ROC and DCA curves. **(G)** ROC curves of the feature genes in the training set. **(H)** ROC curves of the feature genes in the validation set.

We built RA diagnostic column line graph models for the signature genes (BTN3A2, CYFIP2, ST8SIA1, and TYMS) using the Rms package (**Figure 7A**) and evaluated their predictive power using calibration curves. The calibration curves revealed that the difference between the real and predicted RA risks was very minimal, indicating that the column line graph model RA is quite accurate (**Figure 7B**). The correctness of the model may also be confirmed using the ROC curve analysis (**Figure 7C**). The “column line graph” curve is higher than the gray line in decision curve analysis (DCA), and the “BTN3A2, CYFIP2, ST8SIA1, and TYMS” curve implies that patients can benefit from the column line graph model at a high-risk threshold of 0 to 1. The column line graph model provided a greater clinical benefit than the “BTN3A2, CYFIP2, ST8SIA1, and TYMS” curve (**Figure 7D**). Validation in the validation set (GSE48780 and GSE55235) also confirmed these findings (**Figures 7E, F**). To further validate the diagnostic value of BTN3A2, CYFIP2, ST8SIA1, and TYMS, we used receiver operating characteristic (ROC) analysis. BTN3A2 (AUC: 0.841), CYFIP2 (AUC: 0.928), ST8SIA1 (AUC: 0.889), and TYMS (AUC: 0.844) were found to have similar AUC values (**Figure 7G**). The validation datasets (GSE48780 and GSE55235) also corroborated the following findings: TYMS (AUC: 741), BTN3A2 (AUC: 0.858), CYFIP2 (AUC: 0.867), ST8SIA1 (AUC: 0.744) (**Figure 7H**). These findings imply that all major genes are involved in RA.

### Immunological Infiltration in the RA Group and Healthy Controls Using ssGSEA Analysis of Immune Correlation

**Figure 8:**
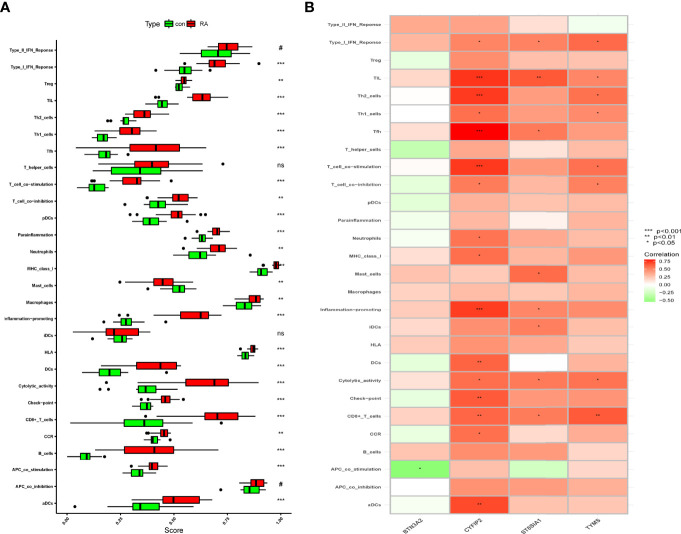
Correlation between RA and immunity. **(A)** Comparison of ssGSEA scores of immune cells and immune pathways between RA group and healthy controls. **(B)** Correlation between characteristic genes and immunity. *p < 0.05, **p < 0.01, ***p < 0.001. NS, no significance.

The immune infiltration association between RA patients and healthy controls was investigated further using ssGSEA. The results showed that immune cell infiltration in mast cells and RA was lower than in the control group after excluding the non-statistical significant ones, and that immune cell infiltration and immune-related pathways in the rest of the RA group were higher than those in the control group (**Figure 8A**). We know that CYFIP2 was associated with aDCs, CCR, CD8+ T cells, check point, cytolytic activity, DCs, inflammation promoting, MHC class I, neutrophils, T-cell co-inhibition, T-cell co-stimulation, Tfh, Th1 cells, Th2 cells, TIL, and Type I IFN response and significantly positively correlated using the “corrplot” package to calculate the correlation between signature genes. BTN3A2 was negatively correlated with APC co-stimulation. CD8+ T cells, cytolytic activity, iDCs, inflammation promoting, Tfh, TIL, and Type I IFN response all had strong positive correlations with ST8SIA1 (**Figure 8B**). These characteristic genes may modulate the immune processes during the progression of RA.

### Increased Expression of CYFIP2 and ST8SIA1 in Synovial Tissues of CIA Mice

**Figure 9:**
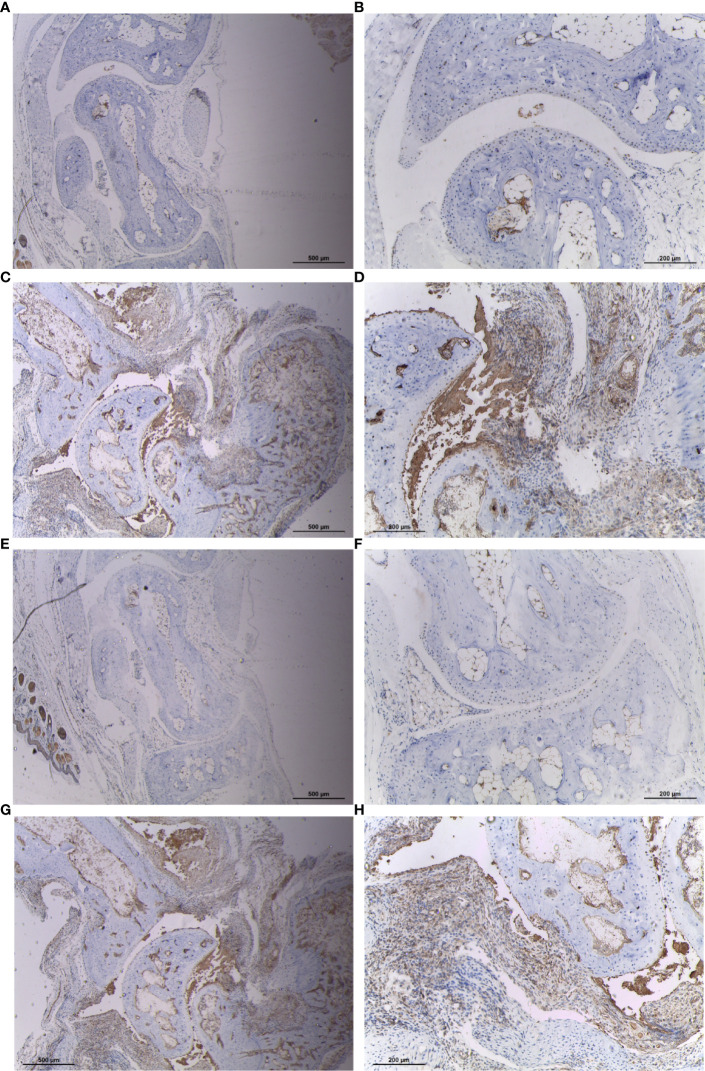
Expression of CYFIP2 and ST8SIA1 in the synovial membrane of CIA mice. **(A, B)** Immunohistochemical analysis of CYFIP2 expression in normal mouse synovium, ((**A**) original magnification ×40, **(B)** original magnification ×100). **(C, D)** Immunohistochemical analysis of CYFIP2 expression in the synovial membrane of CIA mice, ((**C**) original magnification ×40, **(D)** original magnification ×100). **(E, F)** Immunohistochemical analysis of ST8SIA1 expression in normal mouse synovium, ((**E**) original magnification ×40, **(F)** original magnification ×100). **(G, H)** Immunohistochemical analysis of ST8SIA1 expression in the synovial membrane of CIA mice, (**G**) original magnification ×40, **(H)** original magnification ×100).

To verify the expression of CYFIP2 and ST8SIA1 in RA synovium, we treated mouse synovium with IHC and found that CYFIP2 and ST8SIA1CIA mice were highly expressed in the synovium (**Figure 9**).

### Pan-Cancer CYFIP2 Expression

**Figure 10:**
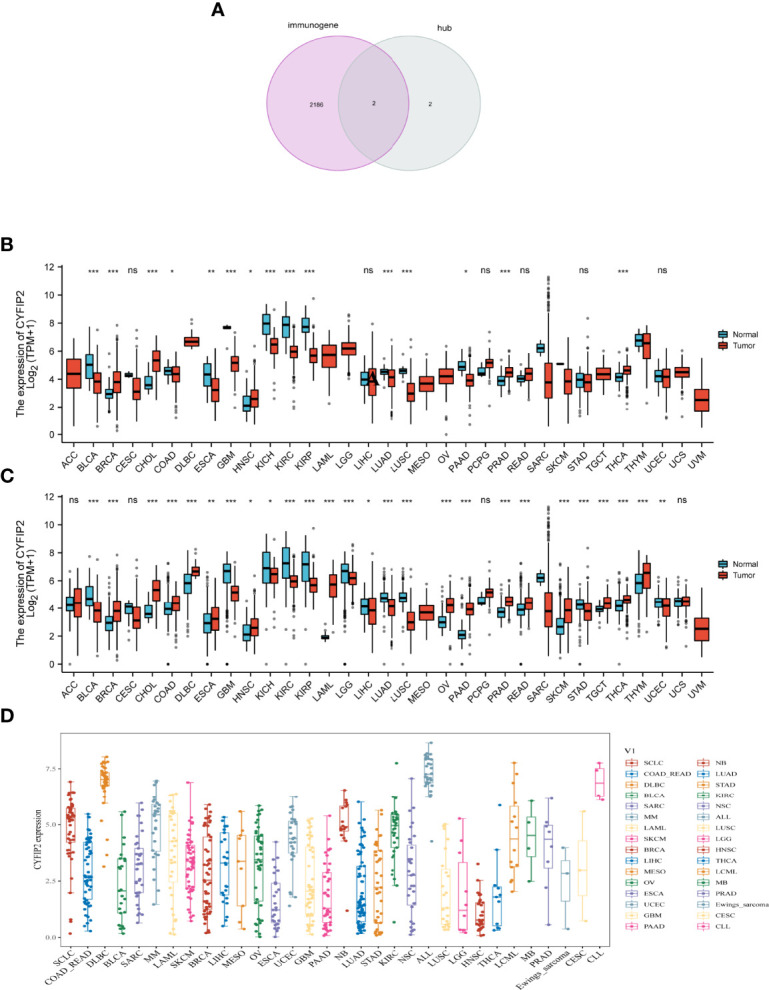
CYFIP2 expression. **(A)** Venn diagram between immunogene and hub genes. **(B)** Pan–cancer expression levels of CYFIP2 in the TCGA dataset. **(C)** Pan–cancer expression levels of CYFIP2 in the TCGA and GTEx datasets. **(D)** Expression of CYFIP2 in various cell lines. *p <0.05, **p <0.01, ***p <0.001. NS, no significance.

Immunity genes were retrieved from the InnateDB database, and four signature genes were crossed to produce two overlapping genes (CYFIP2, ST8SIA1). We took the CYFIP2 gene to the next level of analysis after combining the ssGSEA results. Since the immune response is crucial not only in RA but also in cancer, we used overlapping immune genes to see if there is any link between the two diseases. CYFIP2 was identified to be highly expressed in BRCA, CHOL, HNSC, PRAD, THCA, and low expressed in BLCA, BRCA, COAD, ESCA, GBM, KICH, KIRC, KIRP, LUAD, LUSC, and PAAD in the TCGA data (**Figure 10A**). We also downloaded normal tissue data from the GTEx database and discovered that CYFIP2 was strongly expressed in BRCA, CHOL, COAD, DLBC, ESCA, HNSC, OV, PAAD, PCPG, PRAD, READ, SKCM, TGCT, THCA, and THYM, whereas it was weakly expressed in BLCA, CESC, GBM, KICH, KIRC, KIRP, LGG, LIHC, and LUAD (**Figure 10B**). As demonstrated in the data, CYFIP2 was expressed in the cell lines (**Figure 10C**).

### CYFIP2’s Prognostic Value in Pan-Cancer

**Figure 11:**
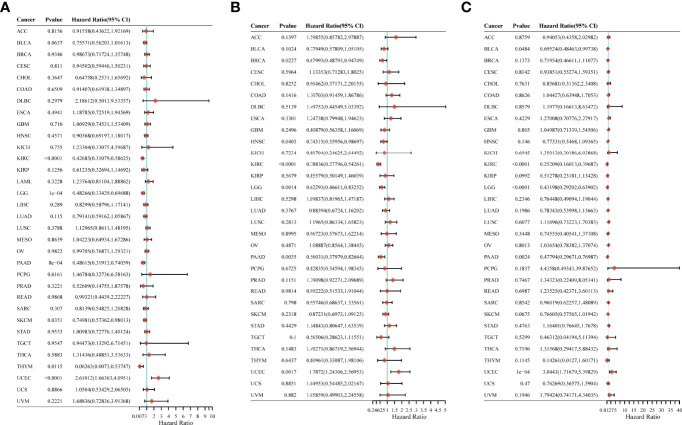
Correlation of CYFIP2 with prognosis in pan–cancer. **(A)** Cox regression model analysis of the correlation between CYFIP2 expression and OS in various tumors. **(B)** Cox regression model analysis of the correlation between CYFIP2 expression and PFS in various tumors. **(C)** Correlation analysis of CYFIP2 expression with DSS in various tumors by Cox regression model.

We looked into the relationship between CYFIP2 expression and pan-cancer patient prognosis, including overall survival (OS), disease-specific survival (DSS), and progression-free survival (PFS). In the OS analysis, cox regression of 33 tumors revealed that CYFIP2 expression was substantially linked with OS in six cancers: KIRC, LGG, PAAD, SKCM, and THYM as protective factors, and UCEC as a risk factor (**Figure 11A**). In the PFS study, cox regression of 33 tumors revealed that CYFIP2 expression was substantially linked with PFS in 6 malignancies, with protective factors in BRCA, HNSC, KIRC, LGG, and PAAD and risk factors in UCEC (**Figure 11B**). In the DSS analysis, Cox regression of 33 tumors revealed that CYFIP2 expression was substantially linked with DSS in 5 cancers: BLCA, KIRC, LGG, and PAAD were protective factors, whereas UCEC was a risk factor (**Figure 11C**).

### Analysis of Immune Infiltration

**Figure 12:**
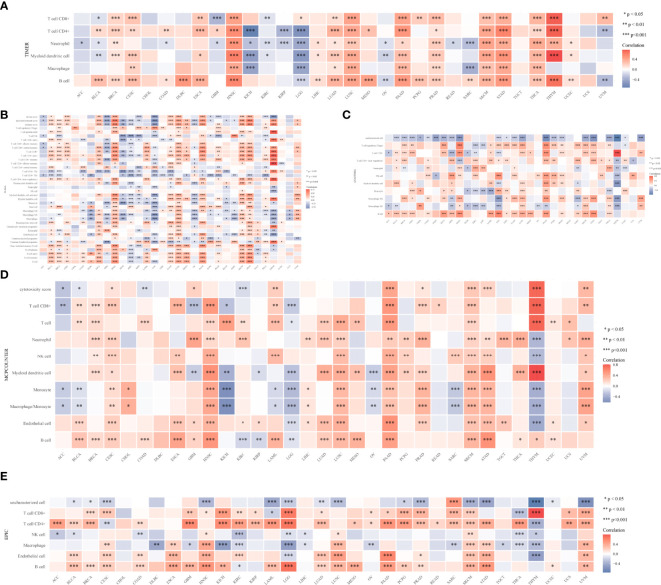
CYFIP2’s role in tumor immune response. **(A)** EPIC_Immu_score. **(B)** XCELL_Immu_score. **(C)** QUANTISEQ_Immu_score. **(D)** MCPCOUNTER_Immu_score. **(E)** TIMER_Immu_score. *p < 0.05, **p < 0.01, ***p < 0.001.

To learn more about the role of CYFIP2 in tumor immune response, the connection between CYFIP2 expression and different levels of immune cell infiltration was calculated using the TIMER database. According to the findings, T-cell CD8+ in 18 tumors, T-cell CD4+ in 20 tumors, neutrophils in 23 tumors, myeloid dendritic cells in 19 tumors, myeloid dendritic cells in 12 tumors, and B cells in 23 malignancies were shown to be strongly connected. HNSC, LUSC, PAAD, SKCM, STAD, THCA, and THYM showed substantial positive correlations, while KICH and LGG showed significant negative correlations (**Figure 12A**). The connection between CYFIP2 levels and invading immune cells was also demonstrated using the xCELL algorithm (**Figure 12B**), the QUANTISEQ algorithm (**Figure 12C**), the MCPCOUNTER algorithm (**Figure 12D**), and the EPIC algorithm (**Figure 12E**). The estimated scores of the stromal score and immune score were calculated using the ESTIMATE algorithm, and the findings revealed that the immune score was related to 13 cancers, while the stromal score was related to 16 tumors. The immunological scores were most closely linked to HNSC (R = 0.64), LGG (R = −0.59), and STAD (R = 0.5) among them. HNSC (R = 0.42), LGG (R = −0.45), and UVM (R = 0.48) had the strongest correlations with the stromal score. CYFIP2 levels and immunological checkpoints were shown to be highly associated in a range of cancers, with mostly positive correlations in UVM and mostly negative correlations in BLCA, BRCA, COAD, HNSC, and PRAD, which were mostly negatively connected in UVM.

### MSI and TMB Analyses

In the TMB study, CYFIP2 was found to be negatively linked with BRCA, COAD, KIRC, LGG, LIHC, LUAD, PAAD, SARC, STAD, THYM, UCEC, and UVM. CYFIP2 was positively and adversely linked with COAD, DLBC, KICH, SARC, and STAD in the MSI analysis (**Supplementary Figure 3**).

## Discussion

Rheumatoid arthritis (RA), a common systematic autoimmune disease, has gained increasing attention around the world recently. The main symptoms of RA include musculoskeletal pain, swollen joints, and stiffness, which can severely impair motor function and quality of life (2). Usually, RA is characterized by inflammation of the tendon, resulting in the destruction of cartilage and bone (23). Clinical data have demonstrated that women, smokers, and patients with a family history are susceptible populations (23), which could not help raise the hypothesis that genes may play a critical role in the pathogenesis. Growing evidence has pointed out that RA is a multi-gene disorder with a substantial genetic component and approximately 60% heritability (3). However, the current common RA symptomatic therapy strategy is conventional disease-modifying antirheumatic drugs (DMARDs), mainly including methotrexate (MTX) and leflunomide (LEF), which are used to reduce inflammation and prevent disease progression (24). The extensive use of DMARDs in worldwide clinical treatment has also made drug-resistance become an issue recently, and new therapy approaches are urgently needed. Therefore, exploring novel genetic targets would provide us with a new insight into RA therapy and treatment strategies.

Recent decades have witnessed the rapid development of molecular research and bioinformatics techniques. By enrichment analysis through molecular function, biological processes, and cellular components, molecular biology could provide us with a comprehensive and further investigation of how gene variation and co-expression influence protein function and disease progression. Meanwhile, the emerging weighted gene co-expression network analysis (WGCNA) has gradually been used in the association between diseases along with related phenotypes and clusters (modules) of highly corrected genes (15). Several studies have illustrated the effects of the hub gene and underlying molecular mechanisms in RA patients through WGCNA analysis (25, 26). While comprehensive immune infiltration and related pathways are still deficient.

Given these, we developed a comprehensive and in-depth evaluation system to analyze and verify hub genes and molecular pathways involved in RA patients through bioinformatics, especially WGCNA and protein–protein interaction (PPI) techniques, aiming to broaden the horizons into physiopathology and molecular mechanisms of RA and provide novel therapeutic targets for clinical treatment.

In this study, we screened 891 differentially expressed genes (DEGs) and found 427 genes were upregulated and 464 were downregulated. Subsequent GO enrichment analysis showed all DEGs mainly associated with cell–cell adhesion, components of the plasma membrane, and cytokine receptor activity, while KEGG enrichment analysis showed some correlation with hematopoietic and T cells, along with chemokine signaling pathways. WGCNA analysis showed 33 cluster samples and 24 modules. No significant correlation verified the reliability of dividing parts. Critical machine algorithms and LAASO regression analysis found 4 hub genes, then validation datasets confirmed that BTN3A2, CYFIP2, ST8SIA1, and TYMS were highly expressed in RA, and the first three genes were highly similar in biological function.

Several research reported a certain association between the 4 hub genes and the process of RA to a certain extent. An article by Horsburgh et al. illustrated that CpG-specific methylation at RA might become a marker of treatment response. Most notably, one of the CpG sites in the BTN3A2 genes was strongly associated with treatment response (27). Actually, as a crucial mediator in immune activation, butyrophilin subfamily 3 member A2 (BTN3A2) was widely investigated in cancer initiation and development, revealing a tight link between immune infiltration and cancer development, especially in breast cancer (BRCA) and ovarian cancer (OV) (28). From a molecular aspect, there was also evidence demonstrated that epithelial BTN3A2 expression was significantly associated with a higher density of infiltration T cells, particularly CD4+ cells (29), which was similar to our enriched outcome at some level. However, the exact prognostic value of BTN3A2 in RA patients still warrants further investigation. Meanwhile, as for the gene CYFIP2, there is also a lack of research about it in the field of RA. A meta-analysis unearthed that CYFIP2 was upregulated and validated in peripheral blood mononuclear cell samples of RA patients, creating a novel gene signature in RA diagnostic and therapeutic interventions (30). An investigation focused on the downregulation of CYFIP2 in clear cell renal cell carcinoma (ccRCC) revealed that several immune markers were critically correlated with CYFIP2 expression, especially with CD4+ cells and CD8+ cells, which could act as a tumor suppressor gene in ccRCC and create a novel strategy in clinical treatment (31). Except for RCC, current research about CYFIP2 was mainly concentrated on neurons and encephalopathy (32, 33), and more attention should be paid to RA. Similar to CYFIP2, most research about ST8SIA1 mainly focused on cancer, revealing that ST8SIA1 regulated tumor growth and metastasis by activating the FAK/AKT/mTOR signaling pathway in breast cancer (34, 35), or inhibited the progression and invasion of bladder cancer cells by suppressing the JAK/STAT signaling pathway (36). However, no evidence was found in the RA research.

Unlike the above three genes, there is already adequate literature about TYMS in RA research. Thymidylate synthase (TYMS) is an important enzyme in the *de novo* pyrimidine pathway responsible for DNA replication (37). To predict the response or toxicity of MTX in patients with RA, a study by Bae et al. conducted a meta-analysis that demonstrated no association between the TYMS polymorphism and non-responsiveness to or toxicity of MTX therapy (38). Another investigation pointed out that polymorphic variations in the TYMS genes indicated a better clinical response to combined DMARD regimens containing MTX (39), and Lima et al. revealed similar results (40). This contrary research means further, more comprehensive and in-depth investigation is warranted to make certain the association between TYMS and MTX.

To illustrate the action of hub genes in RA one step further, we conducted a GSEA analysis, and the results showed that the primary immunodeficiency was significantly enriched in the high-expression subgroup of all 4 hub genes, and the intestinal immune network for IgA was enriched in CYFIP2 high-expression and ST8SIA1 high-expression subgroups, while hematopoietic cell lineage, Th1 and Th2 cell differentiation were enriched in ST8SIA1 high-expression and TYMS high-expression subgroups at the same time. Subsequent ROC analysis showed that all hub genes played a critical role in RA, indicating a potential diagnostic value in clinical treatment.

Finally, further immune infiltration analysis showed the mast cells in the RA group were higher than those in the control group. Mast cells could stimulate osteoclast differentiation in monocytes and then stimulate osteoclastogenesis, which is a mechanism of inflammatory and tissue destruction effects in RA patients (41). The association between hub genes and immune cells was mainly concentrated on CD8+ T cells, inflammation promoting, Tfh, TIL, and type 1 IFN response, which agreed with previous studies (42, 43).

To further explore the core genes among the four key genes, we chose to download immune genes from the InnateDB database and found two overlapping genes (CYFIP2 and ST8SIA1) at the intersection of the four characteristic genes. After *in vitro* validation using RA mice, it was found that the expression levels of both genes were increased, which further confirmed our previous research inferences. Combined with ssGSEA analysis, CYFIP2 was highly correlated with more immune cells and immune response processes compared with ST8SIA1. Therefore, we used CYFIP2 as the target gene for further analysis.

Interestingly, some existing studies point to a relationship between RA and a variety of cancers. On the one hand, RA has been pointed out to have a relationship with the risk of cancer, including lung cancer (44), lymphoma (45), and breast cancer (46, 47), on the other hand, immunosuppressive agents used in RA treatment have also been shown to increase cardiovascular disease and important factors in cancer risk (48, 49). Therefore, we further explored the role of the hub gene found in RA, CYFIP2, in pan–cancer.

In our study, CYFIP2 was a prognostic protective factor for KIRC, LGG, and PAAD, and a risk factor for UCEC, but there is still a lack of relevant studies to prove it. From the overall situation of the current research, CYFIP2 has been studied more in digestive system cancers. For example, the study by Mongroo et al. (50). showed that CYFIP2 is highly expressed in IMP–1 knockdown colon cancer cell lines. This high expression is very important. It may be an important part of preventing CRC tumor cell death, similarly, Vandamme T et al. also found the up–regulation of CYFIP2 in pancreatic cancer (51). In addition, CYFIP2 has also been found to affect lymphoma progression after undergoing epigenetic modifications (52). These studies have fully demonstrated that CYFIP2 plays an important role in human diseases. In addition, pan–cancer–based immune cell infiltration analysis also revealed that CYFIP2 is closely related to T–cell CD8+, T–cell CD4+ and neutrophils. These high infiltrating fractions of cells are consistent with the results we obtained in RA.

Even though this is a comprehensive and novel evaluation system to explore hub genes and related signaling pathways in RA patients, even in pan–cancer, there were also several limitations in our study. Firstly, although we performed validation of gene expression in mice, due to the innate restrictions of bioinformatics techniques, more experiments *in vivo* or *in vitro via* human samples are warranted to confirm our results. Secondly, because our data are from a database, some aspects like sex, age, and complications are not considered in our research, and further clinical investigation is needed.

### Conclusion

To explore specific hub genes for the association between immune infiltration and RA as well as pan–cancer, we conducted a comprehensive and in–depth analysis to analyze related genes and pathways. The 2 hub genes (CYFIP2 and ST8SIA1) we discovered would broaden our insights into molecular mechanisms and bring more potential therapeutic targets for clinical treatment, which also needs more research to verify and develop. For further pan–cancer analysis, CYFIP2 was considered the most potential target both in RA and 33 kinds of tumors, which may shed the hoping light on the therapy of human immune–related diseases and even cancer.

## Data Availability Statement

The original contributions presented in the study are included in the article/supplementary material. Further inquiries can be directed to the corresponding author.

## Ethics Statement

The animal study was reviewed and approved by the Experimental Animal Ethics Committee of Jinan University.

## Author Contributions

ZYZ, SJH, and XCY planned the research concept and designed it, made provisions for study material, collected data and analyzed them, wrote and approved the manuscript. XFL and ST searched for data and wrote programming code. MHW, MMA, and XQH collected pictures and graphs as well as edited them. SZ and DSZ collected data and analyzed them, wrote and approved, and helped correct the manuscript. All authors listed have made a substantial, direct, and intellectual contribution to the work and approved it for publication.

## Funding

This research is funded by the Shenzhen Key Laboratory of Musculoskeletal Tissue Reconstruction and Function Restoration and Shenzhen People’s Hospital (Project number: ZDSYS20200811143752005), the Guangzhou Science and Technology Project (Grant No. 201904010060, Effect and mechanism of S100A4 on collagen–induced arthritis (CIA) model in mice, the National Natural Science Foundation of China (Project number:81401766), the Fundamental Research Funds for the Central Universities (Project number: 21619348), the National Natural Science Foundation of China (Project number: 81901650), and the Science and Technology Projects in Guangzhou (Project number: 2021020200460).

## Conflict of Interest

The authors declare that the research was conducted in the absence of any commercial or financial relationships that could be construed as a potential conflict of interest.

The reviewer Z–WG declared a shared parent affiliation with the author MEA to the handling editor at the time of the review.

## Publisher’s Note

All claims expressed in this article are solely those of the authors and do not necessarily represent those of their affiliated organizations, or those of the publisher, the editors and the reviewers. Any product that may be evaluated in this article, or claim that may be made by its manufacturer, is not guaranteed or endorsed by the publisher.
