# Supplementary material for: Analysis and Experimental Validation of Rheumatoid Arthritis Innate Immunity Gene CYFIP2 and Pan-Cancer
Source: Front Immunol. 2022 Jul 11;13:954848. doi: 10.3389/fimmu.2022.954848 (PMC9311328; doi:10.3389/fimmu.2022.954848)
Supplement: Supplementary file 1 [file DataSheet_1.docx]

Supplementary Material

## Supplementary Figures


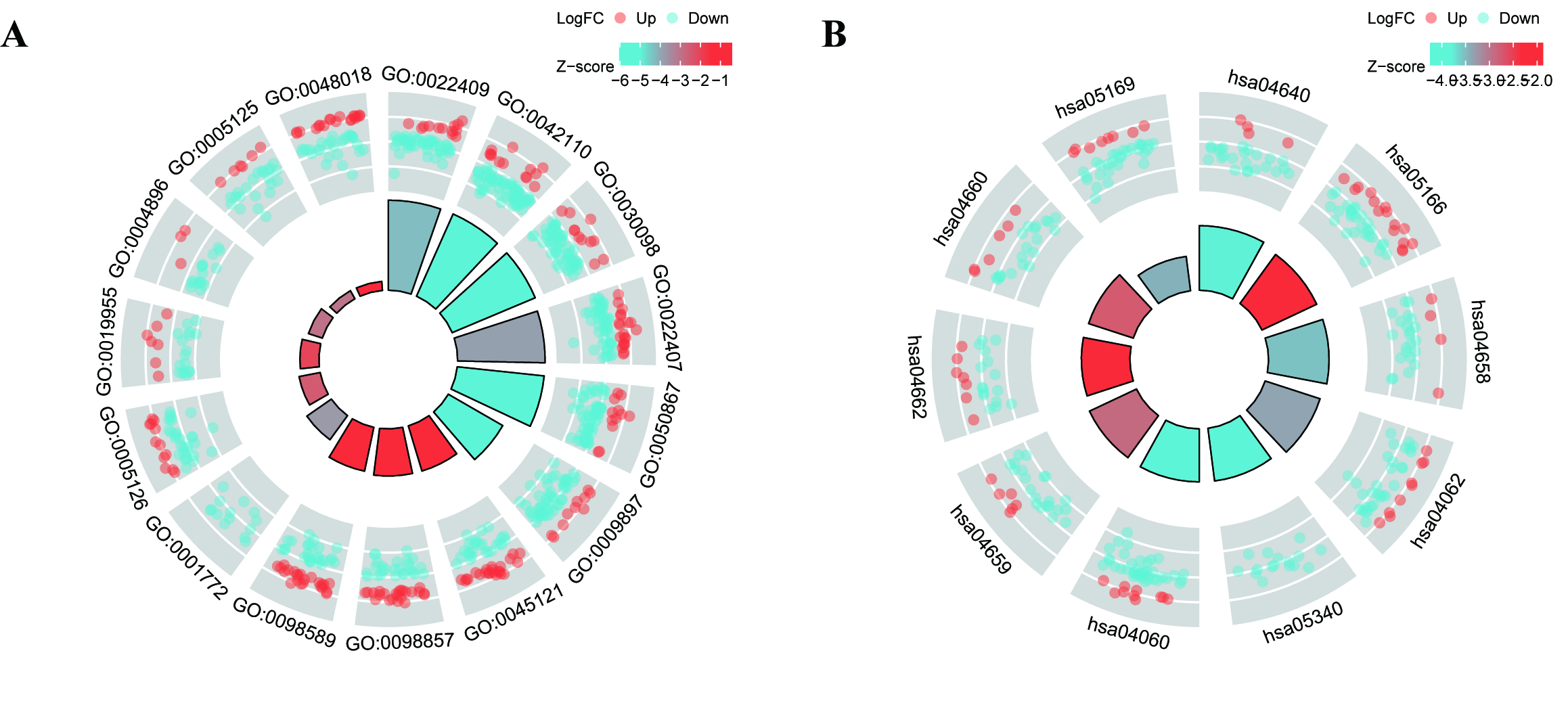


**supplement 1.** Functional enrichment analysis of DEG. (A) GOCircle plot showing 15 GO terms. (B) KEGGCircle plot showing 10 KEGG terms. The height of the bars in the inner ring represents the log10-adjusted P-value of the GO terms, and the higher the bar indicates the higher importance of the GO category, and the color corresponds to the Z-score. descriptions of the GO categories are shown in the table at the bottom


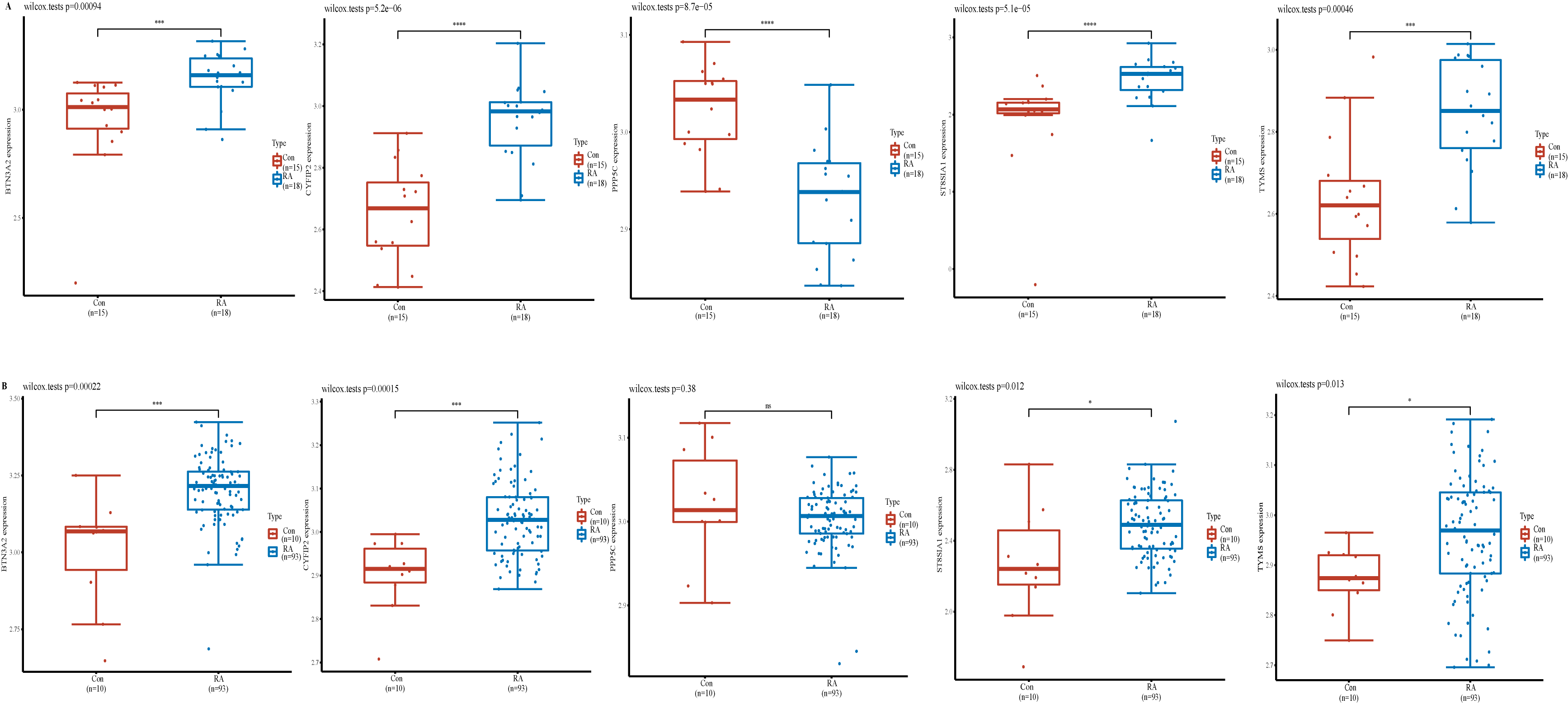


**supplement 2.**Characterized gene expression validation. (A) Feature genes were expressed in GSE1919 and GSE55447 datasets. (B) Feature gene expression in GSE48780 and GSE55235 datasets.


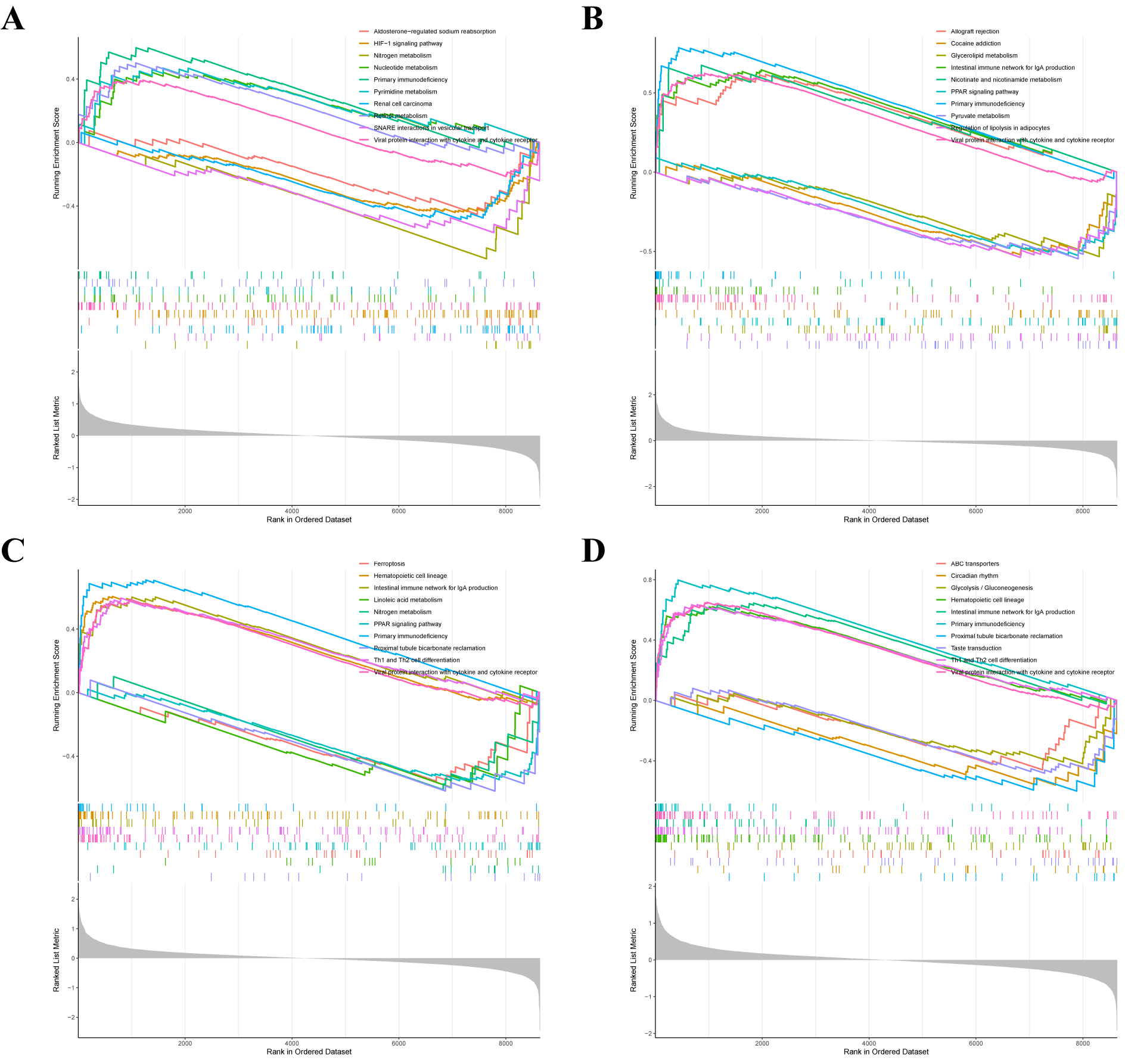


**supplement 3.**GSEA analysis of characteristic genes. (A) GSEA analysis of BTN3A2 gene. (B) GSEA analysis of CYFIP2 gene. (C) GSEA analysis of ST8SIA1 gene. (D) GSEA analysis of TYMS gene.
