# Supplementary material for: Analysis and Experimental Validation of Rheumatoid Arthritis Innate Immunity Gene CYFIP2 and Pan-Cancer
Source: Front Immunol. 2022 Jul 11;13:954848. doi: 10.3389/fimmu.2022.954848 (PMC9311328; doi:10.3389/fimmu.2022.954848)
Supplement: Supplementary file 2 [file Table_1.doc]

| Ontology | ID | Description | GeneRatio | BgRatio | pvalue | p.adjust | qvalue |
| --- | --- | --- | --- | --- | --- | --- | --- |
| BP | GO:0022409 | positive regulation of cell-cell adhesion | 59/857 | 255/18670 | 1.57e-25 | 8.68e-22 | 5.93e-22 |
| BP | GO:0042110 | T cell activation | 80/857 | 464/18670 | 3.96e-25 | 8.71e-22 | 5.95e-22 |
| BP | GO:0030098 | lymphocyte differentiation | 69/857 | 353/18670 | 4.73e-25 | 8.71e-22 | 5.95e-22 |
| BP | GO:0022407 | regulation of cell-cell adhesion | 73/857 | 403/18670 | 2.45e-24 | 3.28e-21 | 2.24e-21 |
| BP | GO:0050867 | positive regulation of cell activation | 72/857 | 394/18670 | 2.97e-24 | 3.28e-21 | 2.24e-21 |
| CC | GO:0009897 | external side of plasma membrane | 62/868 | 393/19717 | 1.47e-18 | 8.69e-16 | 6.90e-16 |
| CC | GO:0045121 | membrane raft | 49/868 | 315/19717 | 1.14e-14 | 2.54e-12 | 2.02e-12 |
| CC | GO:0098857 | membrane microdomain | 49/868 | 316/19717 | 1.29e-14 | 2.54e-12 | 2.02e-12 |
| CC | GO:0098589 | membrane region | 49/868 | 328/19717 | 5.63e-14 | 8.32e-12 | 6.61e-12 |
| CC | GO:0001772 | immunological synapse | 14/868 | 36/19717 | 1.42e-10 | 1.68e-08 | 1.34e-08 |
| MF | GO:0005126 | cytokine receptor binding | 40/853 | 286/17697 | 1.30e-09 | 1.15e-06 | 9.78e-07 |
| MF | GO:0019955 | cytokine binding | 24/853 | 128/17697 | 9.14e-09 | 4.04e-06 | 3.44e-06 |
| MF | GO:0004896 | cytokine receptor activity | 19/853 | 96/17697 | 1.30e-07 | 3.84e-05 | 3.27e-05 |
| MF | GO:0005125 | cytokine activity | 28/853 | 220/17697 | 2.65e-06 | 5.86e-04 | 4.99e-04 |
| MF | GO:0048018 | receptor ligand activity | 47/853 | 482/17697 | 3.62e-06 | 6.41e-04 | 5.45e-04 |
