# Supplementary material for: Analysis and Experimental Validation of Rheumatoid Arthritis Innate Immunity Gene CYFIP2 and Pan-Cancer
Source: Front Immunol. 2022 Jul 11;13:954848. doi: 10.3389/fimmu.2022.954848 (PMC9311328; doi:10.3389/fimmu.2022.954848)
Supplement: Supplementary file 3 [file Table_2.doc]

| Ontology | ID | Description | GeneRatio | BgRatio | pvalue | p.adjust | qvalue |
| --- | --- | --- | --- | --- | --- | --- | --- |
| KEGG | hsa04640 | Hematopoietic cell lineage | 28/551 | 99/8076 | 4.21e-11 | 1.25e-08 | 7.97e-09 |
| KEGG | hsa05166 | Human T-cell leukemia virus 1 infection | 43/551 | 219/8076 | 1.60e-10 | 2.14e-08 | 1.36e-08 |
| KEGG | hsa04658 | Th1 and Th2 cell differentiation | 26/551 | 92/8076 | 2.15e-10 | 2.14e-08 | 1.36e-08 |
| KEGG | hsa04062 | Chemokine signaling pathway | 39/551 | 192/8076 | 4.16e-10 | 3.10e-08 | 1.97e-08 |
| KEGG | hsa05340 | Primary immunodeficiency | 16/551 | 38/8076 | 9.68e-10 | 5.77e-08 | 3.67e-08 |
| KEGG | hsa04060 | Cytokine-cytokine receptor interaction | 50/551 | 295/8076 | 1.16e-09 | 5.78e-08 | 3.67e-08 |
| KEGG | hsa04659 | Th17 cell differentiation | 27/551 | 107/8076 | 1.57e-09 | 6.70e-08 | 4.26e-08 |
| KEGG | hsa04662 | B cell receptor signaling pathway | 23/551 | 82/8076 | 2.84e-09 | 1.06e-07 | 6.72e-08 |
| KEGG | hsa04660 | T cell receptor signaling pathway | 26/551 | 104/8076 | 3.93e-09 | 1.30e-07 | 8.28e-08 |
| KEGG | hsa05169 | Epstein-Barr virus infection | 37/551 | 202/8076 | 2.27e-08 | 6.77e-07 | 4.31e-07 |
